# Management and outcome of patients with traumatic brain injury treated in three major Nordic intensive care units: a comparative cohort study

**DOI:** 10.1186/s13049-026-01657-7

**Published:** 2026-06-26

**Authors:** Hanna Lidström, Anna Hutchens, Logan Froese, David W. Nelson, Jiri Bartek, Alexander Fletcher-Sandersjöö, Dag Ferner Netteland, Mads Aarhus, Cecilia A. I. Åkerlund, Wivi Taalas, Jari Siironen, Juho Vehviläinen, Markus B. Skrifvars, Rahul Raj, Eric P. Thelin, Cathrine Tverdal

**Affiliations:** 1 Department of Clinical Neuroscience, Karolinska Institutet, Stockholm, Sweden; 2 University of Oxford, Oxford, UK; 3 Medical Unit Neurology, Karolinska University Hospital, Stockholm, Sweden; 4 Department of Physiology and Pharmacology, Section of Perioperative Medicine and Intensive Care, Karolinska Institutet, Stockholm, Sweden; 5 Function Perioperative Care and Medicine, Karolinska University Hospital, Stockholm, Sweden; 6 Medical Unit Neurosurgery, Karolinska University Hospital, Stockholm, Sweden; 7 Institute of Clinical Medicine, Faculty of Medicine, University of Oslo, Oslo, Norway; 8 Department of Neurosurgery, Oslo University Hospital, Oslo, Norway; 9 Department of Neurosurgery, Helsinki University Hospital and University of Helsinki, Helsinki, Finland; 10 Department of Anaesthesiology and Intensive Care, Helsinki University Hospital and University of Helsinki, Helsinki, Finland

**Keywords:** Traumatic brain injury, Trauma center, Mortality, Outcome, Neurosurgical intervention, Intensive care unit, Temporal trends

## Abstract

**Background:**

Traumatic brain injury (TBI) is a major cause of death and disability, with large variations in management strategies and outcomes across countries. Nordic countries are similar in demographics, but differences in treatment strategies likely exist and outcome differences have been reported. We aimed to assess temporal differences in treatments and outcomes in three large Nordic centers, with a hypothesis that outcomes, after adjustment for injury severity, are generally similar and have improved over time.

**Materials and methods:**

We performed an international, multicenter, retrospective, observational registry study including consecutive intensive care unit (ICU) managed TBI patients from three of the largest university hospitals in the Nordics (Helsinki (HUS), Finland [2005–2020]; Stockholm (KUH), Sweden [2005–2022]; Oslo (OUH), Norway [2015–2022]). Our primary outcome was 6–12-month unfavorable Glasgow Outcome Scale (GOS, 1–3) and secondary outcome was 30-day mortality. We compared outcomes after adjusting for TBI outcome predictors: age, pupil responsiveness and admission Glasgow Coma Scale (GCS) over time. Other known outcome predictors like computerized tomography (CT) scores (Marshall CT classification and Rotterdam CT score) were collected if available, and intracranial pressure monitoring (ICP) for GCS 3–8 patients was specifically analyzed.

**Results:**

In total, 5,970 patients were included (HUS = 2,769, KUH = 1,390, OUH = 1,811). Six-months crude unfavorable outcome was 43% and predicted unfavorable outcome was 44%. Crude 30-day mortality was 17% and predicted 30-day mortality was 18%. Of the included patients, 72% had a moderate-to-severe TBI (GCS 3–12) and 54% underwent some form of neurosurgical intervention. Model application diminished differences between centers, across both outcome measures. Minimal variation in outcomes between hospitals was observed, with a hospital-level variance after applying the model of 0.02 (SD 0.15) for unfavorable outcome and 0.06 (SD 0.24) for 30-day mortality. Overall, no significant temporal trend in predicted unfavorable outcome was observed (OR per year 0.95, 95% CI 0.88–1.03, *p* = 0.20). Variations in ICP monitoring, but also caseload, were observed.

**Conclusion:**

Differences in patient case-mix and caseload between hospitals were associated with variation in treatment strategies and crude outcomes. However, after adjusting for TBI outcome predictors, no meaningful between-hospital variation in outcomes was observed, and no temporal trends in outcome found over the study period.

**Supplementary Information:**

The online version contains supplementary material available at 10.1186/s13049-026-01657-7.

## Background

Traumatic brain injury (TBI) causes a substantial number of hospitalizations each year and the consequences can be devastating, leading to death or severe disability. TBI affects people of all ages but the trend in high-income countries, such as in the Nordics, reflects an increasing proportion of elderly admitted to hospitals [1]. Nordic countries share similar demography and social culture, and the countries are welfare states with a single-payer publicly funded health care [2]. However, there are also differences, such as geographical size of regions and population density. Through the Scandinavian Neurotrauma Committee (SNC) [3], several guidelines for the management of TBI have been published resulting in many prehospital, neurosurgical and neuro-intensive services adhering to similar guidelines [4–8]. However, there are no established Nordic guidelines for the management of severe TBI [9], instead different variations of Brain Trauma Foundation (BTF) [10] guidelines are generally used, sometimes influenced by the Lund concept [11]. Presumably as an effect of this, studies have shown significant differences in management between the Nordic centers [12–14]. Registry studies in recent years originating from Nordic neurotrauma centers have provided insight in management and outcome after TBI [15–20], but studies are usually limited to single centers or based on national data, which make direct comparison challenging. Furthermore, comparison between centers, or countries, may detect clinically relevant differences, which after being properly addressed could improve patient outcomes.

Worldwide, there are vast differences in outcome following severe TBI, with a systematic review from 2022 suggesting a varying mortality range between 18 and 75% and unfavorable outcome rates ranging from 29 to 100%, with a full recovery seen in 21–27% of patients [21]. The CENTER-TBI project, including 65 centers in Europe, also revealed significant differences between centers, with variations existing in ICU stay and treatment policies as well as outcomes [22]. This highlights that even within or between countries of similar demographic makeup, different outcomes exist. Unfortunately, outcome studies often fail to include adjustments for injury severity, which complicates adequate comparisons between centers, as very different populations and severities may end up being compared [1, 21, 23, 24]. Two previous Finnish studies [25] showed that outcomes in ICU-treated TBI patients, when adjusted for certain outcome predictors have improved with time the last 20 years, which we assume would apply to other Nordic centers as well.

This study combined data from hospital databases and compared patient populations to identify potential differences in treatment strategy and patient outcomes between neurosurgical centers managing moderate-to-severe TBI in Oslo, Stockholm and Helsinki over time. Analyses in a combined dataset may provide a stronger basis for evaluating the quality of trauma care, with the purpose of contributing to the continuous improvement of patient management. Thus, the study aim was to compare the patient and treatment characteristics between three major Nordic trauma centers, assess changes in crude outcomes as a factor of time, and compare predicted outcomes between centers. Our hypothesis is that patient and treatment characteristics vary between centers, and that predicted outcome has improved over time, but that there will be no changes in predicted outcomes between centers.

## Materials and methods

### Study centers and databases

**Fig. 1:**
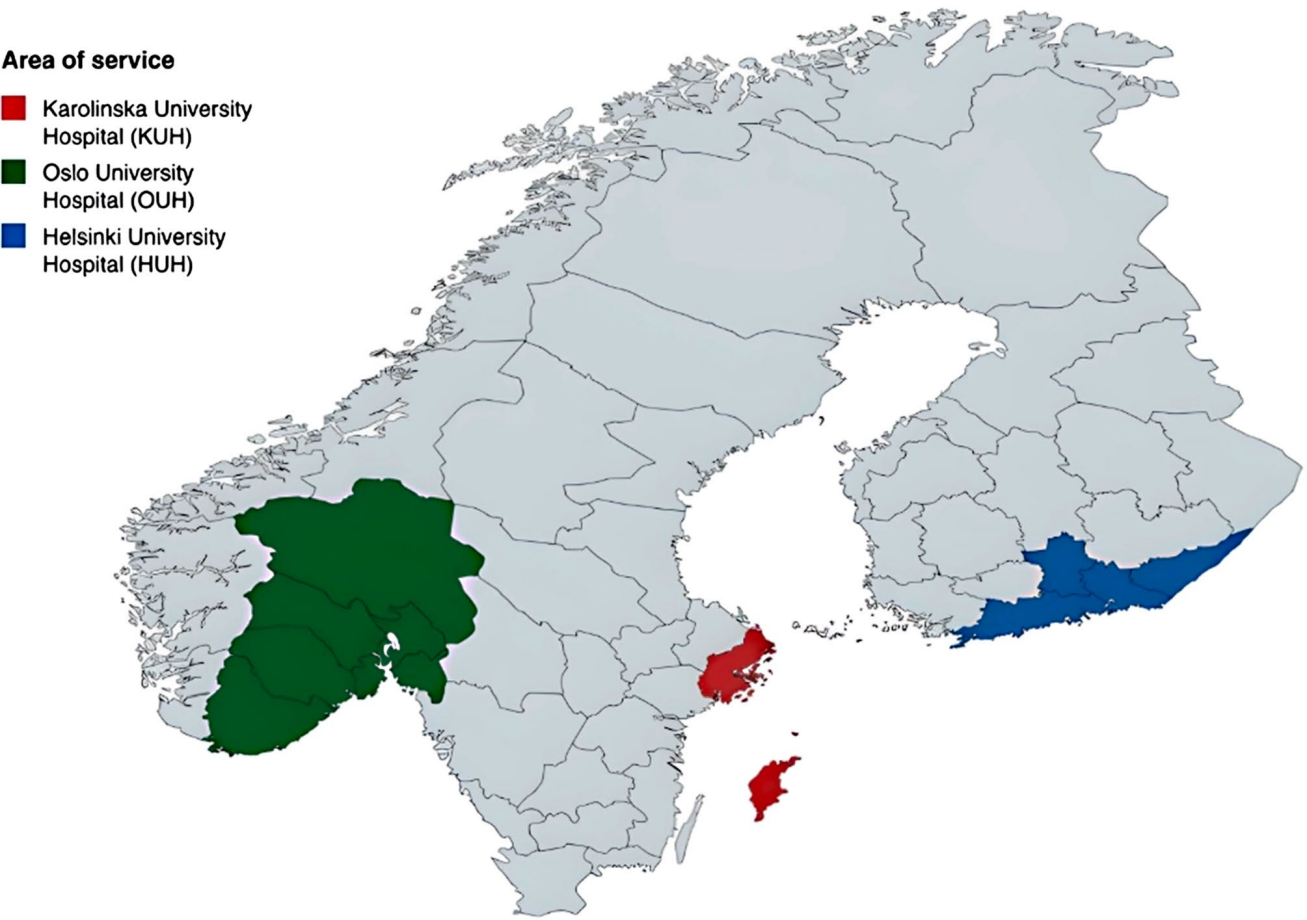
Geographical areas serviced by each hospital

This was an international, multicentre retrospective observational registry-based study. Oslo University Hospital (OUH) in Norway, Karolinska University Hospital (KUH) in Sweden, and Helsinki University Hospital (HUS) in Finland are large specialist neurotrauma centers (university hospitals) in their region. Each of the three centers cover a population in the range of 2.2 to 3 million, but spread over areas of different geographical size: ~6.500 km^2^ (KUH), ~ 9.100 km^2^ (HUS) ~ 110.000km^2^ (OUH) (Fig. 1). Over time, each of the hospitals have established unique TBI quality control databases with the purpose of improving care and facilitating research. In all three registries, patient data is manually retrieved from available information in electronic medical records. The databases include several well-known and established clinical variables and outcome predictors in TBI and trauma care.

### Management protocols and site specific information

#### HUS

Helsinki University Hospital (HUS) is the only Level I trauma center in the region of Southern Finland and covers ~ 9.100 km^2^ and a population of ~ 2.2 million, covering all neuro-intensive care and neurosurgical care.

At HUS, the treatment protocol is based on the BTF [10] and during the later years the SIBICC [26] guidelines with local modifications. Indications for ICP monitoring include unconsciousness (motor score ≤ 5) in conservatively treated TBI with radiological signs of elevated ICP (e.g., midline shift, basal cistern compression, or mass lesion), or persistent unconsciousness (motor score ≤ 5) following hematoma evacuation with residual signs of intracranial hypertension on CT. In cases of evacuated mass lesions without radiological signs of elevated ICP, patients are managed without ICP probes using regular wake-up tests. When ICP is monitored, the treatment target is ICP < 20 mmHg and CPP 60–90 mmHg (in patients with known chronic hypertension, a CPP up to 90 mmHg is tolerated). Primary decompressive hemicraniectomy is considered in salvageable patients where brain edema or midline shift is disproportionately large relative to the hemorrhagic lesion(s), generally in patients under 60 years.

Helsinki TBI Registry is a practical quality control database run by the Department of Neurosurgery. The database is regularly retrospectively updated and contains patients with acute TBI (24 h from trauma to admission) admitted to the neurosurgical ICU since 1999 (12). In total, at the time of data extraction, the registry includes almost 4.000 ICU TBI patients. The functional outcome is registered at 6 months by hospital chart review.

#### KUH

Karolinska University Hospital (KUH) is the only Level I trauma center and the only hospital with neurosurgical services in the Stockholm Region. The Stockholm Region includes 26 municipalities covering ~ 6.500 km^2^ and a population of ~ 2.5 million (including the island of Gotland).

At KUH, we similarly adhere to guidelines similar to BTF and to a large extent the SIBICC guidelines (especially towards the later part of the study period) [10, 26]. Relevant mass lesions are evacuated, and intracranial monitoring inserted, if deemed suitable by the attending neurosurgeon. ICP was usually measured with external ventricular drains (EVD) during the early phase of the study but at about 2015, intraprenchymal pressure devices became the preferred choice (Codman Neuro, DePuy Synthes Companies of Johnson & Johnson, New Brunswick, NJ, USA or Rehau AG & Co, Bern, Germany). ICP is targeted below the threshold of 20 mmHg. The patients are elevated to 30-degree angle with ICP measured at the temple. In case of high ICP, CPP is used to guide management, targeted at 55–70 mmHg, usually target using autoregulatory assessments. CPP is maintained by using vasopressors and intravascular infusions. Unconscious patients are intubated and mechanically ventilated, and anesthetized with opioids, propofol, or midazolam. If patients have a refractory high ICP, barbiturate coma (monitored using burst-suppression on electroencephalogram (EEG)) is commonly initiated, followed by hemicraniectomy if ICP remains high. Primary hemicraniectomy is performed in cases with acute brain swelling at admission surgery, and the autologous bone flap is generally used for later replacement. If traumatic subarachnoid hemorrhage (trSAH) is present, the patient is commonly managed with nimodipine. Body temperature is targeted at around 37 °C without, regulated predominantly with paracetamol, but occasionally during the study period parecoxib or external cooling devices. Cerebral microdialysis and oxygen pressure monitoring devices are used in selected cases, and serial biomarker sampling in blood (S100B) to screen for potential deterioration was used throughout the study period [27, 28].

Stockholm TBI Registry is a quality control database run initially by the Department of Neurosurgery and then subsequently by the Department of Anesthesiology (Funktion PMI) at KUH. Since 2005 the database is more systematically structured, especially concerning outcome. Patients are prospectively registered, and data is manually retrieved from available information in electronic medical records. The registry includes patients admitted to KUH with verified computerized tomography (CT) / magnetic resonance imaging (MRI) findings. Since 2018, fewer mild-to-moderate patients are managed locally at KUH as more focus is on severe TBI and complex trauma patients. In total, at the time of data extraction, the registry includes ~ 1.500 ICU TBI patients.

#### OUH

Oslo University Hospital (OUH) is a Level I trauma center and the only hospital with neurosurgical services in the South-East region in Norway. The South-East region covers ~ 110.000km^2^ and a population of ~ 3 million. OUH is also the trauma referral center for the population in Oslo (~ 700.000).

The treatment protocol for TBI at OUH follows the BTF and SIBICC guidelines with local modifications. The recommendations are available for clinicians in a dedicated handbook and an online resource [29]. At OUH, patients with severe TBI (GCS < 9) are routinely admitted. Patients with GCS 9–14 and CT abnormalities and/or deteriorating GCS are generally eligible, but admission is decided on a case-by-case basis by the on-call neurosurgeon and the trauma team leader.

Concerning management, in brief, the indications for ICP monitoring are: (1) GCS < 9 and pathological CT, (2) GCS < 9 and ≥ 2 of the following: age > 40 years, systolic BP<90mmHg, GCS motor < 4 on the best side, (3) GCS < 13 and long-lasting surgery in other organ systems, or expected long-lasting ventilator treatment due to injuries in other organ systems than CNS. Target thresholds for ICP guided therapy in adults are CPP>60mmHg and ICP<22mmHg. The MAP transducer is placed at the level of the tragus. The indications for craniotomy and evacuation of mass lesions are left to the discretion of the neurosurgeons on call, however, a written algorithm based on GCS, lesion volume, midline shift and hematoma width is available to guide the clinicians. Decompressive hemicraniectomy may be considered if there is persistent ICP>22mmHg despite all other NICU measures, there is failure of ICP reduction despite evacuation of mass lesion, and if CT images and the clinical situation is compatible with a meaningful outcome and age < 60 years.

“Oslo TBI Registry – Neurosurgery” is a quality control database run by the Department of Neurosurgery at OUH since 2015. Patients are prospectively registered, and data is manually retrieved from available information in electronic medical records. The registry includes patients admitted to OUH with verified CT/MRI findings of acute trauma (hematoma/hemorrhage, fracture, traumatic axonal injury, vascular injury). In total, at the time of data extraction, the registry includes ~ 3.500 patients, out of which ~ 1.800 are admitted to ICU.

### Inclusion

Patients were eligible for inclusion if above 15 years old and admitted to intensive care units (ICUs) within 24 h from sustaining a TBI. As to make sure that two sites were always compared, we included patients from the year of 2005 to the year of 2022 when data from a minimum of two centers was available. Patients were excluded if missing data regarding Glasgow Coma Scale (GCS) at either the scene of accident or arrival at the hospital or the ICU or missing Glasgow Outcome Score (GOS) for assessing outcome.

Patients before 2005 and after 2022 were excluded in order to ensure overlap between sites. At OUH, due to the number of patients admitted for observation with very short stays in the ICU, an extra criterion was applied: patients were excluded if they had both GCS ≥ 13 and an ICU stay under 24 h. These patients were counted under the ‘not admitted to the ICU’ criterion.

### Clinical variables

The choice of adjustable parameters were restricted to common variables between sites, or common near collinear variables such as CT scores [23]. These included age, sex, GCS, pupillary reflex, CT scores (Marshall CT Classification [30], available at KUH and HUS, and Rotterdam CT score [31], available at KUH and OUH). We also included data on intracranial surgery and if intracranial pressure (ICP) monitoring was present. Due to differences in data collection procedures between centers, variable definitions were compared between sites to identify potential incongruity. For this manuscript, this applied specifically to GCS. The GCS scores were those recorded upon arrival at the specialized hospital. If this data was not available, GCS scores at the primary hospital, or if also missing, scores at the scene of accident were used [32]. Pupillary reflexes were defined as none, one or two pupils without light reflex. Both OUH and KUH have many more variables available in their databases as compared to HUS, so the current data presented represents what was collected across all three sites.

### Outcome

The primary outcome was unfavorable functional outcome (GOS 1–3) at 6–12 months after injury, stated as 180 days unfavorable outcome or mortality. As secondary outcome, 30-day mortality was used. At OUH and HUS functional outcome is registered at 6 months by hospital chart review, at KUH functional outcome is registered at 3–6 months by chart review and at 12 months by standardised questionnaires.

### Statistical analysis

We compared differences in patient characteristics, treatment-related factors and outcomes by producing p-values using two-sided chi-square tests for categorical variables and the Kruskal-Wallis test for continuous non-parametric data and t-test for normally distributed data. We created predicted outcomes by adjusting for statistically significant differences in patient characteristics and treatment-related variables between centers using a logistic regression model (lrm) with covariates of age, GCS and pupil responsiveness to assess their association with outcome. Hospital was included as the primary independent variable, and outcome was modelled as a binary dependent variable. Pseudo-R² values (Nagelkerke R²) were reported to quantify the explanatory power of the models, and their components. From the fitted model, odds ratios for each hospital (with 95% confidence intervals) were obtained, representing the risk of the given outcome after controlling for covariates. Between-hospital variation in outcomes was assessed using mixed-effects logistic regression with hospital as a random intercept, adjusting for age, GCS, and pupil reactivity (lme4 package in R). Temporal trends were estimated by including year as a fixed effect. The resulting coefficient represents the predicted change in odds per year for the given outcome variable, controlling for age, GCS, pupil responsiveness, and hospital. To assess the predictive value of CT imaging on outcomes, a separate logistic regression analysis was performed with Marshall CT classification and Rotterdam CT score as independent variables and outcome as the dependent variable. Models were fitted overall and stratified by hospital. The entire analysis was conducted using R (version 2025.05.1 + 513) scripts.

## Results

### Patient characteristics

**Fig. 2:**
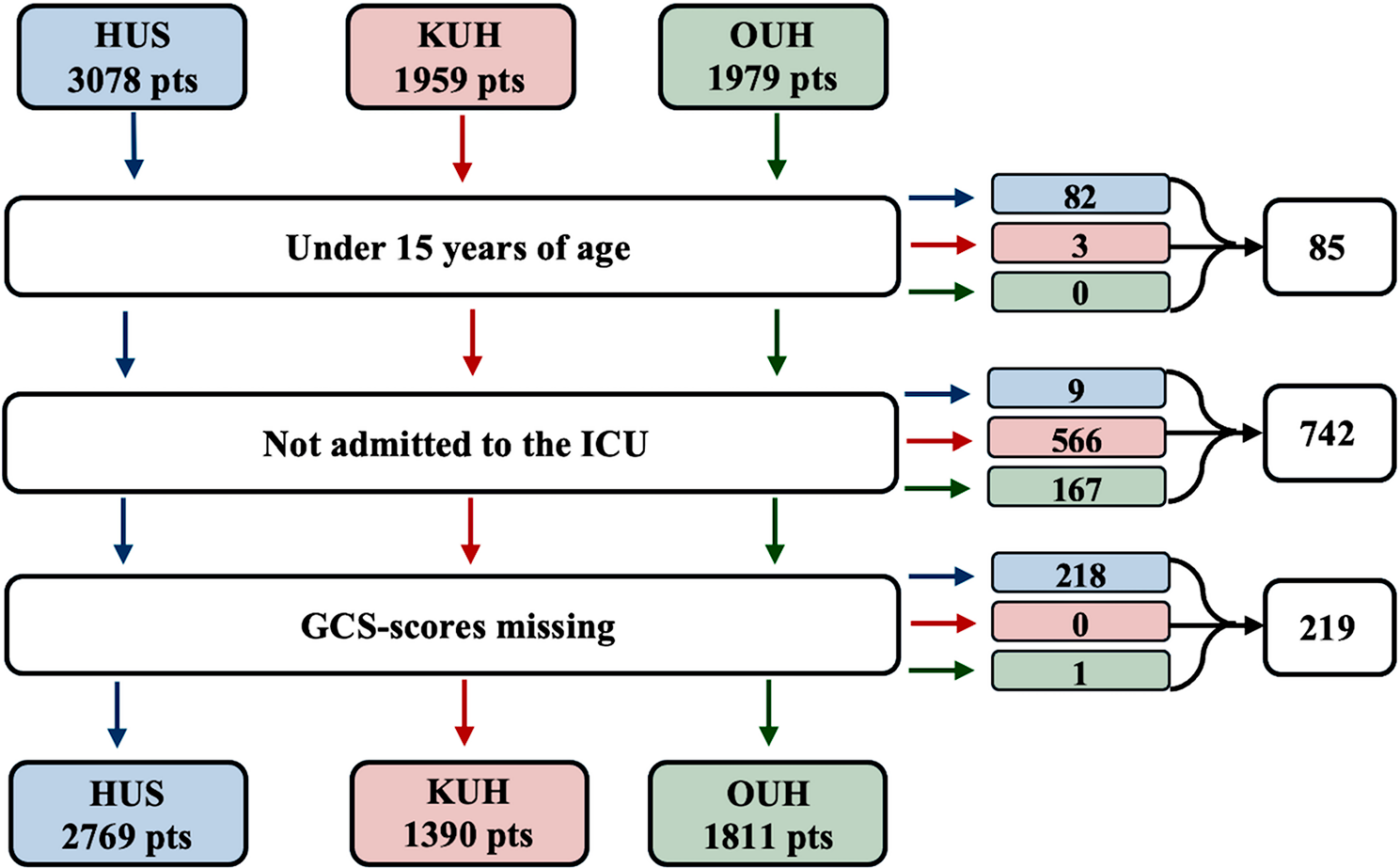
Summarises the results of exclusion criteria application to the three cohorts. The numbers to the right represent the number of missing from each site during the different steps

Inclusion flow-chart is described (Fig. 2). As is shown, this cohort consist of TBI patients in need of ICU care for their injuries. After applying the inclusion criteria, there were no missing GOS at KUH and OUH. However, *n* = 180 patients from HUS had missing GOS. These were excluded from analyses requiring GOS. As for the specific inclusion criteria for OUH, there were *n* = 4 patients in the dataset with admission GCS ≥ 13 and that died within 24 h which are included in the study.

The length of stay at the ICU differed across sites, with KUH having the longest length of stay with a median at 6 days (IQR 2–14), compared to HUS at 4 days (IQR 2–9) and OUH at 3 days (IQR 2–9) (Table 1). Trauma mechanism also differed between sites, with HUS having a higher proportion of falls from ground level (57%) and lower proportion of road traffic accidents (12%) than KUH (38% and 34% respectively) and OUH (43% and 26% respectively). Neurosurgical intervention occurred in a higher proportion of patients at KUH, with a particularly notable difference in proportion of patients receiving ICP monitoring (68% KUH, 17% HUS and 34% OUH). Decompressive craniectomy (9% KUH, 4% HUS and 4% OUH) also occurred in a higher proportion of patients at KUH. Craniotomy occurred with at notably higher frequency in HUS (46%) and KUH (48%) than OUH (21%). In total, the share of patients who received any kind of neurosurgical intervention (including monitoring) was 73% at KUH, 54% at HUS and 40% at OUH. However, it should be noted that the annual number of surgeries was different, with HUS and OUH performing over 50% more surgeries annually as compared to KUH, which corresponded to the lower patient load at KUH (77 per year) which is less than half than for the other centers, suggesting a different patient selection and cohort for Stockholm.

**Table 1:** Descriptive data of patient cohort, injury characteristics and outcome

Years (average per year)	HUS (*n* = 2,769)	KUH (*n* = 1,390)	OUH (*n* = 1,811)	Total (*n* = 5,970)
	2005–2020 (173/year)	2005–2022 (77/year)	2015–2022 (226/year)	
**Patient baseline characteristics**				
Age, median (IQR)	57 (42–68)	52 (33–65)	59 (41–73)	57 (40–68)
Sex, male	74%	76%	72%	74%
GCS at hospital admission, median (IQR)	12 (6–14)	7(3–12)	10 (6–14)	10 (4–14)
Moderate to Severe TBI, GCS 3–13 (n)	64% (1,767)	86% (1,199)	75% (1,350)	72% (4,316)
Unilateral Pupil Dilation (n)	11% (314)	6% (80)	7% (124)	9% (518)
Bilateral Pupil Dilation (n)	10% (270)	14% (199)	5% (98)	9% (567)
Marshall CT-classification (IQR)	5 (2–5)	5 (2–5)	-	
Rotterdam CT-score (IQR)	-	3 (3–4)	3 (2–4)	
**Injury Mechanisms**				
Fall from ground	57%	38%	43%	48%
Road traffic accident	12%	34%	26%	21%
Other	31%	28%	31%	31%
**Treatment characteristics**				
Length of stay ICU (IQR)	4 (2–9)	6 (2–14)	3 (2–9)	
Requiring ventilator support (n)	63% (1,742)	84% (1,171)	60% (1,077)	67%
ICP monitoring (n)	17% (463)	68% (808)	34% (621)	34%
Craniotomy surgery (n)	46% (1280)	48% (671)	21% (388)	39%
Decompressive craniectomy (n)	4% (117)	9% (130)	4% (68)	5%
Any neurosurgical intervention	54% (1,500)	73% (1,013)	40% (729)	54%
**Outcomes**				
180-day unfavorable outcome (GOS 1–3)	45%	47%	41%	43%
30-day mortality	19%	14%	17%	17%
180-day mortality	26%	19%	22%	23%
**Predicted Outcomes (after adjustment for age**,** GCS and pupil responsiveness (with 95% confidence intervals))**				
180-day unfavorable outcome (GOS 1–3)	44% (CI: 43.1–44.9)	43% (CI: 42.1–44.6)	46% (CI: 44.7–46.9)	44% (CI: 43.8–45.0)
30-day mortality	17% (CI: 16.2–17.4)	17% (CI: 16.1–17.7)	20% (CI: 19.3–20.8)	18% (CI: 17.4–18.2)
180-day mortality)	23% (CI: 22.1–23.4)	22% (CI: 21.4–23.3)	25% (CI: 24.6–26.3)	24% (CI: 23.1–24.0)

Any neurosurgical intervention included craniectomies/hemicraniectomy and/or ICP-monitoring. CI = confidence intervals, IQR = interquartile range, TBI = traumatic brain injury, GCS = Glasgow Coma Scale, GOS = Glasgow Outcome Scale, ICU = Intensive care unit, ICP = Intracranial pressure, HUS = Helsinki University Hospital, KUH = Karolinska University Hospital, OUH = Oslo University Hospital

### Functional outcome at 6–12 months

The overall average crude unfavorable functional outcome (GOS 1–3) was 45% at HUS, 47% at KUH and 41% at OUH (Table 1). The parameters age, pupil responsiveness and GCS in a combined model explained variance of between 0.316 (KUH) and 0.407 (OUH) Nagelkerke pseudo-R^2^ (Table 2).

**Table 2:** Outcome analyses

Predictor:	HUS		KUH		OUH		All	
	*R*²	Odds Ratio (95% CI)	*R*²	Odds Ratio (95% CI)	*R*²	Odds Ratio (95% CI)	*R*²	Odds Ratio (95% CI)
**Unfavorable Functional Outcome**								
Age + GCS + Pupil reactivity	0.320	NA	0.316	NA	0.407	NA	0.342	NA
Age (OR per year)	0.143	1.05 (1.04–1.06)	0.156	1.05 (1.04–1.06)	0.212	1.06 (1.05–1.07)	0.165	1.05 (1.05–1.06)
GCS (OR per point)	0.058	0.87 (0.85–0.89)	0.053	0.88 (0.85–0.91)	0.071	0.84 (0.81–0.87)	0.065	0.87 (0.85–0.88)
Pupillary reactivity (OR per pupil)	0.041	2.35 (1.97–2.81)	0.053	2.23 (1.82–2.73)	0.064	4.94 (3.52–6.95)	0.050	2.63 (2.33–2.98)
Marshall CT Classification (OR per point)	0.073	1.51 (1.41–1.62)	0.012	1.11 (1.06–1.18)	NA	NA	0.029	1.22 (1.17–1.27)
Rotterdam CT Score (OR per point)	NA	NA	0.055	1.50 (1.37–1.66)	0.193	2.39 (2.15–2.66)	0.123	1.92 (1.79–2.06)
**30-Day Mortality**								
Age + GCS and Pupil reactivity)	0.283	NA	0.299	NA	0.470	NA	0.322	NA
Age (OR per year)	0.066	1.04 (1.03–1.04)	0.098	1.05 (1.04–1.06)	0.139	1.06 (1.05–1.08)	0.097	1.05 (1.04–1.05)
GCS (OR per point)	0.033	0.89 (0.87–0.92)	0.037	0.86 (0.82–0.91)	0.068	0.81 (0.78–0.85)	0.040	0.87 (0.86–0.89)
Pupillary reactivity (OR per pupil)	0.077	2.73 (2.32–3.22)	0.090	2.49 (2.03–3.05)	0.132	6.69 (4.89–9.16)	0.092	3.02 (2.69–3.38)
Marshall CT Classification (OR per point)	0.094	1.64 (1.48–1.82)	0.006	1.08 (1.0-1.16)	NA	NA	0.045	1.29 (1.22–1.37)
Rotterdam CT Score (OR per point)	NA	NA	0.094	1.65 (1.44–1.90)	0.386	2.96 (2.62–3.35)	0.246	2.32 (2.12–2.54)

Nagelkerke’s pseudo-R^2^ and odds ratios for the different outcome predictors per hospital. HUS=Helsinki University Hospital, KUH=Karolinska University Hospital, OUH=Oslo University Hospital. GCS=Glasgow Coma Scale. CT= Computerized Tomography. OR=Odds ratio. Unfavorable outcome defined as Glasgow Outcome Score (GOS) 1–3 at 6–12 months after injury

**Fig. 3:**
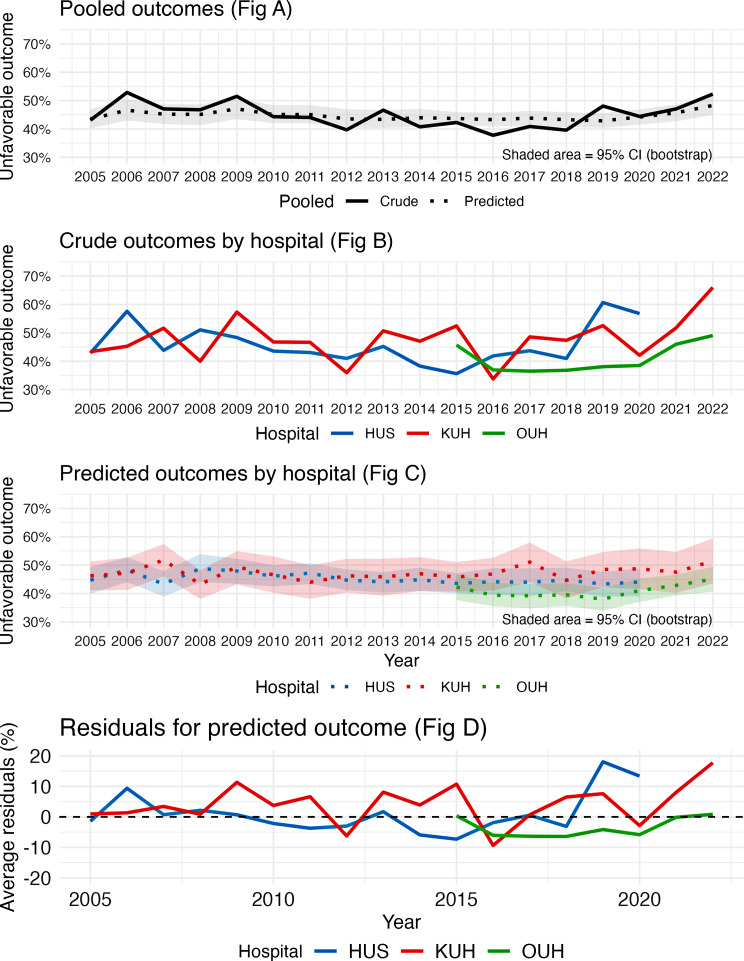
Graph showing unfavorable functional outcome over time combined across the three sites (**A**), as well as crude unfavorable functional outcome (**B**) as well as predicted unfavorable functional outcome after adjustment for age, GCS and pupil responsiveness (**C**) and residuals for predicted unfavorable functional outcome (**D**). HUS = Helsinki University Hospital, KUH = Karolinska University Hospital, OUH = Oslo University Hospital

As observed in Fig. 3 and as can be seen in Supplementary Figs. 1–3, Tables 1, 2 and 3, there is no clear change in either predicted or crude unfavorable outcome over time (Fig. 3A-B). Over the study period, there was no significant temporal trend in predicted 180-day unfavorable outcome (OR per year 0.95, 95% CI 0.88–1.03, *p* = 0.20) (Fig. 3C). No individual site demonstrated a statistically significant change in unfavorable outcome. Comparing crude and predicted outcomes for each site, the crude rate of unfavorable outcome at HUS showed no difference to what would be predicted based on case mix (crude/predicted = 1.01, CI = 0.95–1.07, *p* = 0.71) (Fig. 3D). KUH showed slightly higher crude unfavorable outcome than predicted (crude/predicted = 1.09, CI = 1.00–1.17, *p* = 0.039) and OUH slightly lower (crude/predicted = 0.92, CI = 0.86–0.99, *p* = 0.026).

### 30-day mortality

The crude 30-day mortality was 19% at HUS, 14% at KUH and 17% at OUH (Table 1). The same parameters (age, GCS, pupils) explained 0.322 on average of the estimated explained variance.

**Fig. 4:**
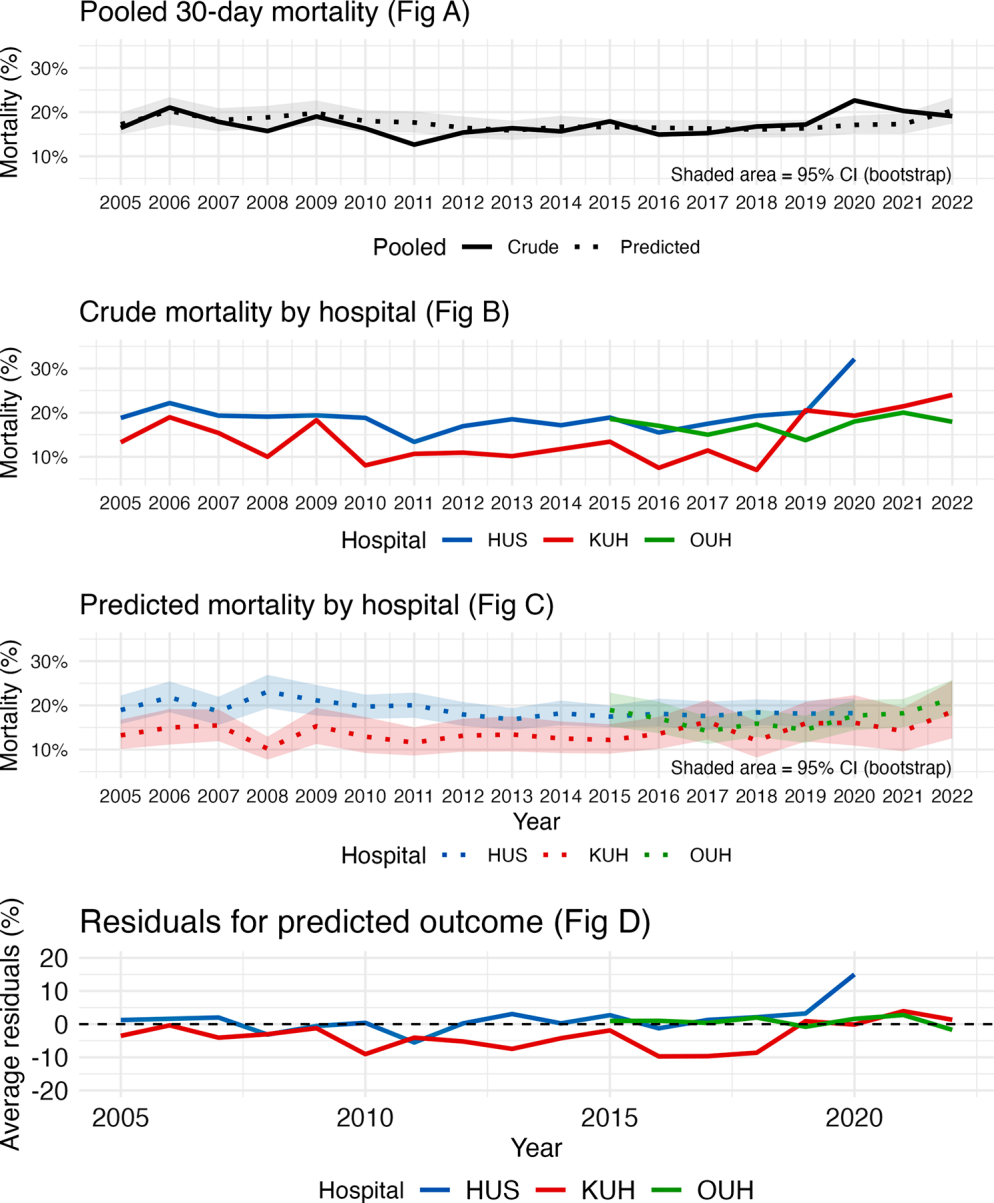
Graph showing 30-day mortality over time combined across the three sites pooled (**A**), as well as crude 30-day mortality (**B**), predicted 30-day mortality after adjustment for age, GCS and pupil responsiveness (**C**) and residuals comparing crude and predicted 30-day mortality (**D**). HUS = Helsinki University Hospital, KUH = Karolinska University Hospital, OUH = Oslo University Hospital

As seen in Fig. 4 crude analyses and Supplementary Figs. 1–3, Tables 1, 2, 3 and 30-day mortality remained stable over time across all hospitals. Over the study period, predicted 30-day mortality appeared to increase over time (OR per year 1.14, 95% CI 1.03–1.25, *p* = 0.009), driven primarily by Helsinki (OR per year 1.20, 95% CI 1.06–1.36, *p* = 0.005), with no significant trends in Oslo or Stockholm (Fig. 4A-B). However, this finding was attributable to an outlier year (2020) in which mortality in Helsinki rose from approximately 20% to 30% (Fig. 4B). If excluding this year, the trend was no longer significant (OR per year 1.06, 95% CI 0.96–1.18, *p* = 0.24). Comparing crude and predicted 30-day mortality for each site, the crude rate of 30-day mortality at OUH showed no difference to what would be predicted based on case mix (crude/predicted = 1.03, CI = 0.92–1.15, *p* = 0.63) (Fig. 4C-D). KUH showed markedly lower predicted 30-day mortality than expected (crude/predicted = 0.78, CI = 0.67–0.89, *p* < 0.001) and HUS higher than expected (crude/predicted = 1.10, CI = 1.01–1.19, *p* = 0.039) (Fig. 4D).

### Variation between hospitals

Mixed-effects logistic regression with hospital as a random intercept revealed minimal between-hospital variation in outcomes. The predicted hospital-level variance was 0.02 (SD 0.15) for 180-day unfavorable outcome and 0.06 (SD 0.24) for 30-day mortality, with corresponding hospital odds ratios of 1.36 (95% CI 0.55–1.82) and 1.63 (95% CI 0.38–2.60), respectively.

### Changes in patient cohort over tine

In the study period there was an increase in patient age over time, with each additional year over the study period being associated with a 4% increase in the odds of being above the median age (OR = 1.04, 95% CI 1.03–1.05, *p* < 0.001) (Supplementary Tables 1–3).

### Changes in ICP-monitoring over time

**Fig. 5:**
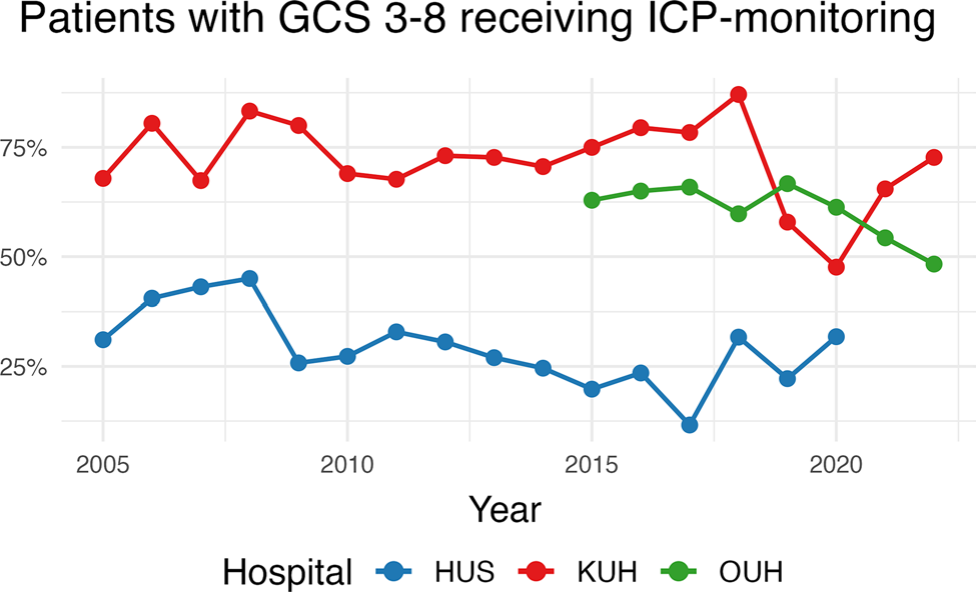
Graph showing changes in the proportion of severe TBI patients receiving ICP monitoring over time. GCS = Glasgow Coma Scale, ICP = Intracranial pressure. HUS = Helsinki University Hospital, KUH = Karolinska University Hospital, OUH = Oslo University Hospital

Even among patients with comparable injury severity, it can be seen that there are marked inter-hospital differences in the use of ICP monitoring (Fig. 5). Among patients with severe TBI (GCS 3–8), 75% at KUH, 61% at OUH, and 30% at HUS received ICP monitoring (overall 53%) (Supplementary Table 4). Similarly, variability persisted in the moderate (GCS 9–12) and mild (GCS 13–15) groups, where the proportion of patients monitored ranged from 16 to 62% and 5–51%, respectively. These differences were even more pronounced when looking at patients with Marshall CT 5–6, where 17% received ICP monitoring at HUS, while 71% received ICP monitoring at KUH (Supplementary Table 5). Among patients with severe TBI, the incidence of ICP monitoring declined significantly over time at HUS (OR per year 0.94, 95% CI 0.92–0.97, *p* < 0.001), and OUH (OR per year 0.92, 95% CI 0.87–0.98, *p* = 0.014), remained stable at KUH (OR per year 0.98, 95% CI 0.95–1.02, *p* = 0.315), and showed an overall decreasing trend after adjustment for center (OR per year 0.96, 95% CI 0.94–0.98, *p* < 0.001).

## Discussion

This study compared patient characteristics, management, and outcomes across three Nordic university hospitals, using harmonised TBI registry data. It is, to the best of our knowledge, the first study with a granular outcome description for crude and predicted outcomes for three Nordic TBI centers. We hypothesised that while inter-center differences in both outcome and management exist, these would be diminished after adjusting for case severity which was largely confirmed by our findings, as if adjusted for known outcome predictors, GCS, age and pupil reactivity, the differences diminish between centers. We further hypothesized that outcomes had improved over the observed time-period, however, crude outcomes did not improve over time (but rather worsen, though not significantly), which could be due to changes in trauma dynamics or an increasing age seen in all three countries, despite our best efforts to adjust for this.

Hence, our first hypothesis, that adjusting outcomes for known outcome predictors would make outcomes more similar across sites was correct. This finding supports the importance of severity adjustment when benchmarking trauma care. This is of particular importance as trauma severity and local traditions/guidelines may change over time, and if not adjusted, policymakers and other key stakeholders may make decisions in resource prioritisation based on an incorrect perception of temporal trends. Further to this, there were some interesting temporal trends observed (such as in ICP-monitoring in severe cases), but this serves to show that the effects of major changes in a healthcare system or guideline implementation and compliance can be monitored using this type of analysis. The number of treated patients at KUH, is notably lower than in HUS and OUH, suggesting a more selective admission criteria: all have similar catchment areas in terms of population, but KUH treats 77 patients/year compared to 173 and 226 patients per year in HUS and OUH, respectively. This is also evident from the more severe case-mix at KUH (Table 2). In the HUS and OUH regions, the university hospitals are the only hospitals treating patients with TBI requiring not only neurosurgical care but also ICU monitoring, which is seen in the older populations in HUS and OUH as compared to KUH. The care of TBI patients is more centralised in OUH and HUS as compared to KUH, where patients are managed at local trauma centers (Trauma Level 2 equivalent) to a larger degree if they do not need immediate neurosurgery or specialised neuro-ICU care for their injuries, which could explain these discrepancies.

Regarding our second hypothesis, that outcomes have improved over time, this could not be concluded based on our data. While in the last 150 years, TBI unfavorable outcomes have decreased substantially [33], they have remained largely unchanged in the past 10 years. Previous Finnish studies have seen an improvement when comparing time periods before 2005 with later years [16, 25], and the mortality at HUS was higher at that time (1995–2004). This is similar to North American studies which shows that while outcomes have improved over time, the largest improvement was during the late 1990s and early 2000s with little or no improvement after that [34]. While there was temporal variation between outcomes, there was no consistent improvement or change in crude or predicted outcomes over time. Apart from annual changes in the case-mix, the steady increase in age, with each additional year over the study period being associated with a 3% increase in the odds of being above the median age, could explain these changes as age is one of the strongest predictors of unfavorable outcome [35]. As age is one of the parameters that we attempt to adjust for in the model, there are likely other reasons that may explain this such as frailty, comorbidities, access to rehabilitation facilities and infrastructural/organisational changes.

When comparing the analysed Nordic centers to European counterparts as seen in CENTER-TBI, both functional outcomes (43% CENTER-TBI and 44% Nordics unfavorable) and mortality rates (21% CENTER-TBI and 24% Nordics) are similar at 6-months [36], but slightly better than e.g. Korean, Chinese and American centers [37–39]. The disparity in outcomes, particularly in comparison to low-and-middle-outcome centers can likely be explained by the fact that these Nordic centers are both resource intensive and higher-income. Also, of note regarding outcomes is that treatment and selection biases may play a role, as in some cases only patients deemed likely to benefit from neurosurgical intervention and intensive care are admitted. Consequently, patients with poor prognostic indicators, such as advanced age or fixed dilated pupils, may be excluded from both treatment and local registries [40], even if favorable outcome in these cohorts are possible with an aggressive treatment approach [41, 42]. Furthermore, at both the HUS and OUH TBI registries, patients are to a larger extent admitted for potential organ donation (POD) as compared to KUH (which started doing this more after legislative changes in 2022). The inclusion of PODs naturally biases both these centers toward higher mortality rates. Future benchmarking must account for this, particularly in Finland, where high organ donation rates, driven largely by neurosurgical patients, lead to PODs comprising a significant portion of ICU deaths [43]. A similar issue arises with treatment limiting decisions, where withholding neurosurgery or the withdrawal of life-sustaining therapies early may also inflate observed mortality rates [44], data which is unfortunately not included in these registries but needs to be integrated into the model for future prospective studies.

The proportion of estimated explained variance differed across hospitals, both for functional outcome and mortality, with OUH data showing higher predictive power compared to the other sites. It is difficult to explain why OUH was so different in terms of ability of the models to estimated explained variance, but possible explanations include treatment biases, or the longer transportation distances/times seen in Oslo, which may result in poorer outcomes [45, 46]. However, in modern cohorts transportation times usually show no or very limited association with outcome [47, 48], so this finding warrants future studies. Rotterdam CT score also showed a substantially higher ability to estimate explained variance at OUH as compared to KUH, reflecting a better ability to distinguish between the full spectrum of patients admitted to OUH, with this granularity not working as well with the more severely injured patients admitted to KUH. A similar effect has been previously observed between Helsinki and Stockholm [13].

KUH had the lowest 30-day mortality across the three centers, but long-term functional outcomes (both crude and predicted) were similar to the other sites. This is notable when considering that these patients were on average more injured (lower GCS and more affected pupils). Perhaps due to the fact that on average patients in KUH were younger and likely had less comorbidities, escalated care was provided independent of the initial prognosis.

Marked inter-hospital differences were observed in ICP monitoring practices. Of note, none of the centers fully adhered to BTF recommendations, which stipulate that all severe TBI patients (GCS 3–8) should have ICP monitoring inserted. While KUH had the highest rate among these patients (75%), the results for all sites are within similar ranges to those observed in the CENTER-TBI study comparing European sites (where a median 61.7% of GCS 3–8 patients had ICP monitors placed) [36]. Another notable difference between hospitals concerns the large differences in absolute numbers and surgeries performed annually. The differences in total numbers, frequency and cases per year are true for all types of neurosurgical interventions as seen in Table 1, rendering it difficult to compare the usefulness or impact on outcomes between sites. When further stratifying patients by severity, management patterns remain heterogeneous. Among GCS 3–8 patients and patients with Marshall CT scores 5–6 (focal mass lesions), HUS had lower rates of ICP monitoring but similar rates of other neurosurgical interventions (e.g. craniotomies and hemicraniectomies), while KUH had the highest proportion receiving intervention. Despite the significant differences in proportion of patients receiving ICP monitoring across sites, this did not seem to affect outcome. Analysis also showed an overall decreasing trend in ICP monitoring among patients with severe TBI (OR per year 0.96, 95% CI 0.94–0.98, *p* < 0.001). This may reflect an increasing number of elderly patients where perhaps on-call teams defer from surgery or a comprehensive clinical appraisal in which extubation and sedation weaning are deemed more appropriate therapeutic strategies.

These findings highlight that even within similarly severe patient groups, inter-hospital management practices differ considerably, likely reflecting different local protocols, varying guideline adherence or a tentative selection bias between patients selected for intensive care. When comparing the whole cohort across sites to CENTER-TBI, where on average 38.6% of patients in the ICU strata had intracranial surgery, the frequency of surgery was notably higher in these centers (ranging from 40 to 73% of patients across sites). This would suggest that neurosurgical intervention being required is one important criterion when considering transfer to KUH where distances are relatively short [39], where patients can instead stay at primary hospitals and are transferred only if they deteriorate. However, due to longer transfer distances in OUH and the healthcare system in Finland, more TBI patients with a lower severity are transferred to these trauma centers for observation.

This study highlights the value, potential but also challenges of standardised, registry-based comparisons across centers, particularly highlighting the importance of adjusting for known outcome predictors. This underscores the need for high-quality data in registries and further improvement in data harmonisation and collaborative benchmarking across sites, but also understanding regional differences and patient selection, to improve evidence-based practice and hospital organisation. One such avenue for improvement are initiatives such as the Nordic University Hospital Alliance (NUHA [49]) and the European Union’s development of the European Health Data Space [50].

### Limitations

There are certain limitations with this study. The three centers do not fully overlap across the entire study period; however, at least two centers contribute data at all time points. While core variables present in e.g. The IMPACT calculator for the core models were present (age + pupils + GCS), imaging scoring varied by site, not allowing us to adjust for them when comparing across all sites, nor was adjustment for any extended labs possible (e.g. glucose, protein biomarkers of brain injury and haemoglobin) nor prehospital information (e.g. hypoxia or hypotension), though the IMPACT core model is a validated severity adjustment model [51, 52]. Due to the retrospective nature of this study, without knowledge of treatment intention and restrictions, it is difficult to draw conclusions as to why results and trends from local sites vary. A complete-case analysis, without imputations, was used as the amount of missing data was limited for the included parameters. However, some variables like GCS which is often not missing at random (and were missing in some patients at HUS), are more affected by this. Moreover, GOS was assessed at 6 months in HUS and OUH, compared to a majority at 12 months in KUH. This longer follow-up period may contribute to a higher rate of favorable outcomes at KUH, as it includes a longer recovery phase [38, 53, 54]. Differentiating between GOS 3 and 4 can also be difficult retrospectively based on patient records. It should also be mentioned that KUH primarily uses approved GOS questionnaires (and only chart reviews if these are missing) as compared to HUS and OUH which conduct hospital charts reviews. Hospital charts reviews are of course dependent on adequate data being present, and have a greater tendency to miss out on daily functioning and social interactions which often require specific questions, thus validated questionnaires are recommended, but are of course more expensive and time consuming [55]. We do believe however that the centralized health care structures of the Nordic countries are unlikely to miss relevant factors for crude outcome assessments, and thus it is possible to compare especially dichotomized GOS between the sites. Also of note is the application of an extra exclusion criterion to OUH to attempt to control for patients admitted for observation: at KUH and HUS, patients are only admitted to the ICU with treatment intention. This does not constitute a major limitation, as if applied to HUS no further patients would be excluded, and at KUH, only *n* = 13 further patients would be excluded. Furthermore, the inclusion of PODs in both the HUS and OUH datasets naturally biases both these centers toward higher mortality rates. For example in HUS, approximately 15–25 patients are admitted to the neuro-ICU due to organ donation per year. Given that PODs account for almost 10% of ICU deaths in Finland, despite representing less than 1% of admissions [43], and their mortality is nearly 100%, this group may constitute a substantial proportion of early TBI deaths. Future registry and benchmarking studies should account for this distinct population. Finally, the cohorts are relatively old which could affect treatments due to frailty and comorbidities [56, 57]. Detailed frailty and/or comorbidity scores are required to better adjust for this, but were unfortunately not available for all centers and were thus not included. Though, as these are severe TBI patients that received intensive care, we do not believe that extensive frailty/comorbidity were present and thus did not significantly affect our results.

## Conclusion

When comparing three major Nordic trauma centers between 2005 and 2022, patients and treatment characteristics varied significantly, but no evidence of improved outcomes over time after adjustment for known predictors, and no major outcome differences between centers, were seen. Crude outcomes fluctuated more than predicted outcomes for each site, highlighting the need to adjust for known TBI outcome predictors even when comparing sites with similar populations. Further prospective research with high-quality data is warranted to better facilitate comparison of outcomes between sites.

## Supplementary Information

Below is the link to the electronic supplementary material.

Supplementary Material 1

Supplementary Material 2

Supplementary Material 3

Supplementary Material 4

## Data Availability

The datasets are not publicly available due to local legal restrictions. Data from KUH may be available through Karolinska Instituet Research Data Office (RDO).

## Abbreviations

TBI: Traumatic brain injury
ICU: Intensive care unit
HUS: Helsinki University Hospital
KUH: Karolinska University Hospital
OUH: Oslo University Hospital
GOS: Glasgow Outcome Scale
GCS: Glasgow Coma Scale
CT: Computed tomography
ICP: Intracranial pressure
SD: Standard deviation
OR: Odds ratio
CI: Confidence interval
SNC: Scandinavian Neurotrauma Committee
BTF: Brain Trauma Foundation
IQR: Interquartile range
lrm: Logistic regression model
POD: Potential organ donor
NUHA: Nordic University Hospital Alliance
