# Supplementary figures and images for: Management and outcome of patients with traumatic brain injury treated in three major Nordic intensive care units: a comparative cohort study
Source: Scand J Trauma Resusc Emerg Med. 2026 Jun 26;34:111. doi: 10.1186/s13049-026-01657-7 (PMC13309966; doi:10.1186/s13049-026-01657-7)

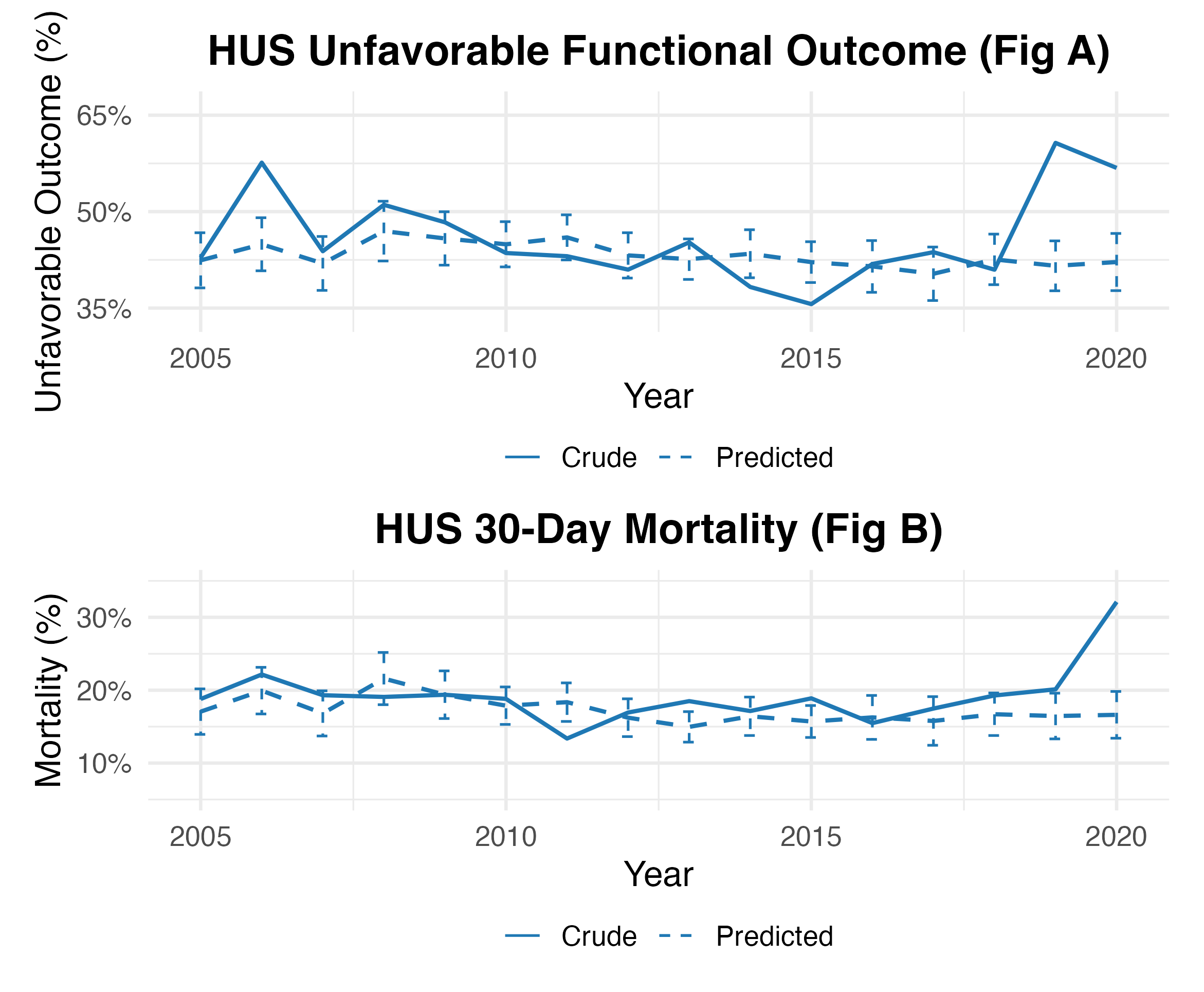

Supplement: Supplementary file 1 — Supplementary Material 1 [file 13049_2026_1657_MOESM1_ESM.tiff]

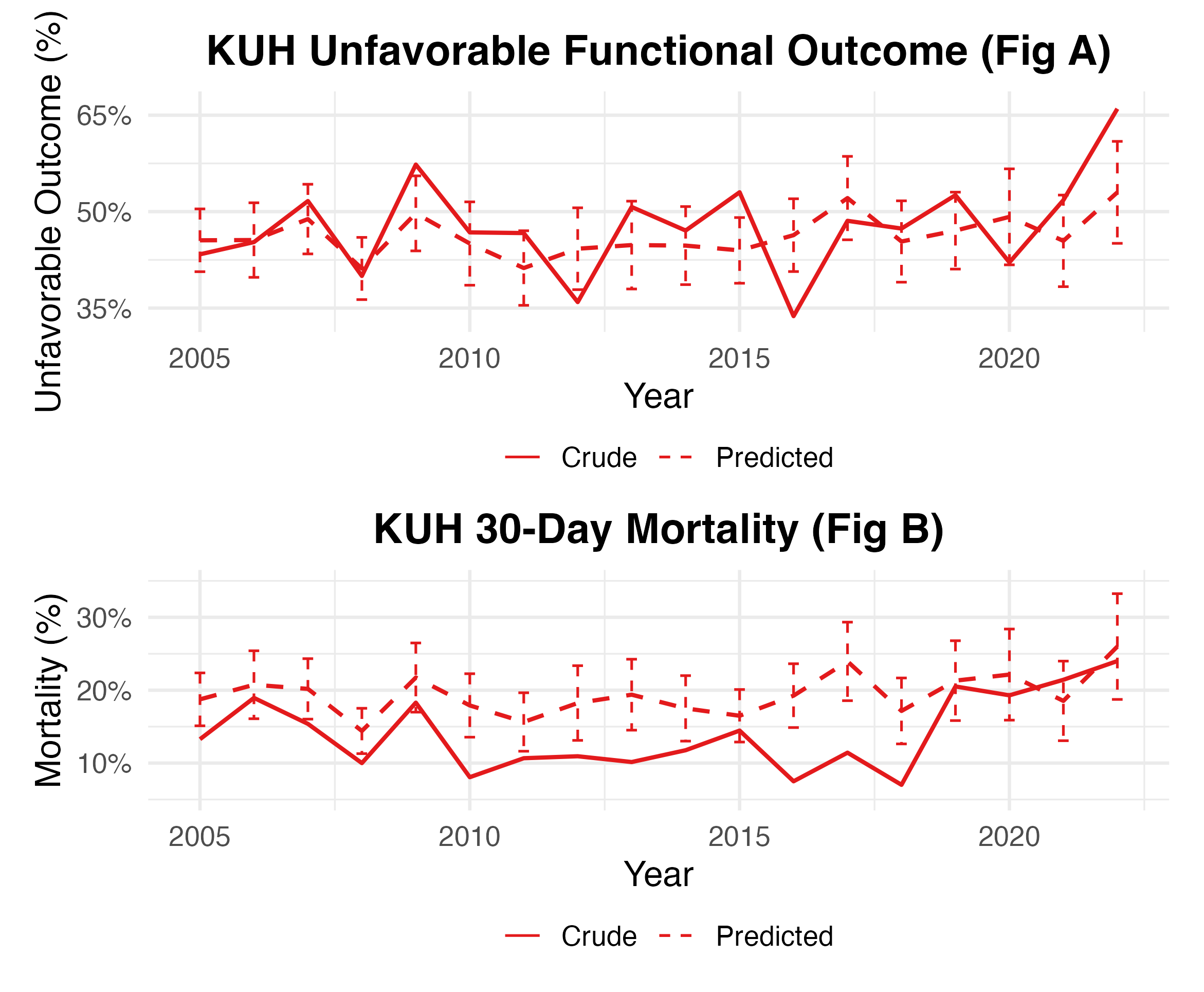

Supplement: Supplementary file 2 — Supplementary Material 2 [file 13049_2026_1657_MOESM2_ESM.tiff]

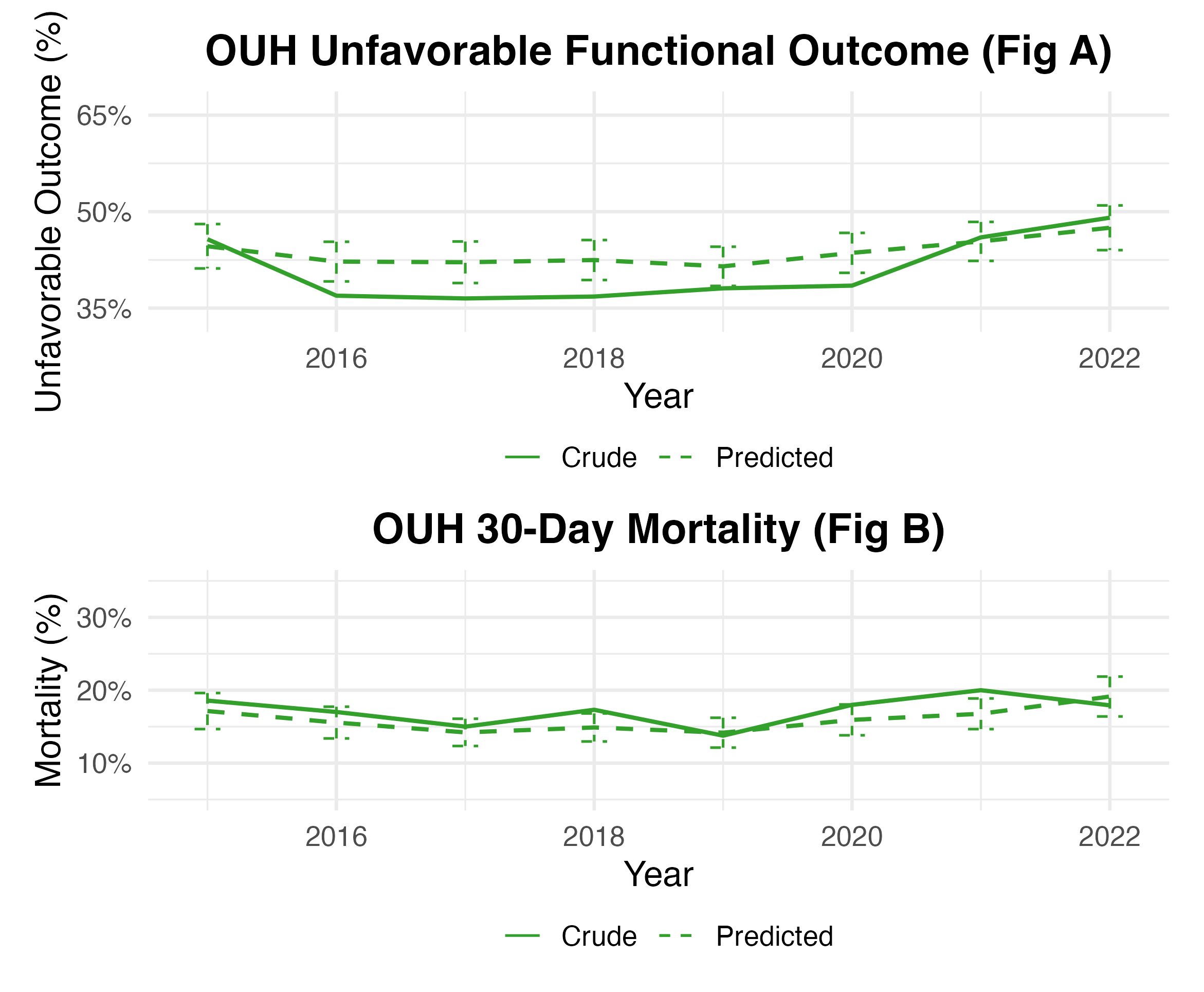

Supplement: Supplementary file 3 — Supplementary Material 3 [file 13049_2026_1657_MOESM3_ESM.tiff]
