# Supplementary material for: Management and outcome of patients with traumatic brain injury treated in three major Nordic intensive care units: a comparative cohort study
Source: Scand J Trauma Resusc Emerg Med. 2026 Jun 26;34:111. doi: 10.1186/s13049-026-01657-7 (PMC13309966; doi:10.1186/s13049-026-01657-7)
Supplement: Supplementary file 4 — Supplementary Material 4 [file 13049_2026_1657_MOESM4_ESM.docx]

Supplementary Table 1 - Differences per year for patients managed at HUS

| **HUS** | **2005** | **2006** | **2007** | **2008** | **2009** | **2010** | **2011** | **2012** | **2013** | **2014** | **2015** | **2016** | **2017** | **2018** | **2019** | **2020** |
| --- | --- | --- | --- | --- | --- | --- | --- | --- | --- | --- | --- | --- | --- | --- | --- | --- |
| Age | 52.2 | 49.0 | 50.3 | 50.6 | 54.4 | 53.3 | 56.2 | 57.0 | 56.1 | 56.8 | 55.5 | 55.2 | 55.6 | 55.7 | 57.5 | 56.1 |
| GCS 3–8 | 40.9 % (61) | 47.2 % (84) | 44.5 % (65) | 52.3 % (80) | 41.2 % (66) | 42.9 % (88) | 40.0 % (82) | 33.2 % (62) | 36.3 % (74) | 36.3 % (65) | 34.0 % (81) | 32.9% (51) | 30.1% (43) | 36.1% (60) | 27.4% (45) | 32.1% (44) |
| GCS 9–12 | 16.8 % (25) | 22.5 % (40) | 19.2 % (28) | 15.7 % (24) | 20.6 % (33) | 18.5 % (38) | 17.1 % (35) | 16.6 % (31) | 14.7 % (30) | 12.8% (23) | 13.4 % (32) | 12.9% (20) | 17.5% (25) | 16.9% (28) | 20.1% (33) | 12.4% (17) |
| GCS 13–15 | 42.3 % (63) | 30.3 % (54) | 36.3% (53) | 32.0 % (49) | 38.1 % (61) | 38.5 % (79) | 42.9 % (88) | 50.3 % (94) | 49.0 % (100) | 50.8 % (91) | 52.5 % (125) | 54.2% (84) | 52.4% (75) | 47.0% (78) | 52.4% (86) | 55.5% (76) |
| Unresponsive pupil/pupils | 21.5 % (32) | 31.8 % (56) | 24.1 % (35) | 34.2 % (52) | 23.8 % (38) | 24.8 % (50) | 23.4 % (48) | 19.1 % (35) | 20.5 % (41) | 15.4 % (27) | 18.5 % (43) | 18.7% (29) | 12.6% (18) | 16.9% (28) | 16.5% (27) | 18.2% (25) |
| Unfavorable Outcome | 42.9% (57) | 57.5% (88) | 44.3% (58) | 51.4% (73) | 48.3% (73) | 43.4% (89) | 42.9% (88) | 40.6% (76) | 45.3% (92) | 38.0% (68) | 35.7% (85) | 41.9% (54) | 43.7% (52) | 41.0% (59) | 60.7% (88) | 56.8% (71) |
| 30-Day Mortality | 18.8% (28) | 22.5% (40) | 19.9% (29) | 19.0% (29) | 19.4% (31) | 19.0% (39) | 13.7% (28) | 17.1% (32) | 19.1% (39) | 16.8% (30) | 18.9% (45) | 15.5% (24) | 17.5% (25) | 19.3% (32) | 20.1% (33) | 32.1% (44) |
| n total | 149 | 178 | 146 | 153 | 160 | 205 | 205 | 187 | 204 | 179 | 238 | 155 | 143 | 166 | 164 | 137 |

Table showing Helsinki University Hospital (HUS) patients within different Glasgow Coma Scale (GCS) categories as well as pupil responsiveness, 6-12 months unfavorable outcome and 30-day mortality, stratified by year.

Supplementary Table 2 - Differences per year for patients managed at KUH

| **KUH** | **2005** | **2006** | **2007** | **2008** | **2009** | **2010** | **2011** | **2012** | **2013** | **2014** | **2015** | **2016** | **2017** | **2018** | **2019** | **2020** | **2021** | **2022** |  |
| --- | --- | --- | --- | --- | --- | --- | --- | --- | --- | --- | --- | --- | --- | --- | --- | --- | --- | --- | --- |
| Age | 48.8 | 47.6 | 53.1 | 49.2 | 48.7 | 47.1 | 49.0 | 50.7 | 46.1 | 49.1 | 48.8 | 49.6 | 50.3 | 47.1 | 50.4 | 53.6 | 50.7 | 52.3 |  |
| GCS 3–8 | 62.7 % (74) | 59.6 % (59) | 62.2 % (61) | 55.4 % (51) | 70.9 % (61) | 61.5 % (40) | 56.6 % (43) | 47 % (31) | 62.0 % (44) | 63.8 % (44) | 50.0 % (42) | 62.4 % (53) | 67.6 % (48) | 68.4 % (39) | 61.5 % (48) | 58.6 % (34) | 66.1 % (37) | 68.6% (35) |  |
| GCS 9–12 | 16.9 % (20) | 18.2 % (18) | 12.2 % (12) | 18.5 % (17) | 12.8 % (11) | 15.4 % (10) | 19.7% (15) | 22.7 % (15) | 14.1 % (10) | 14.5 % (10) | 20.2 % (17) | 18.8 % (16) | 14.1 % (10) | 17.5 % (10) | 23.1 % (18) | 19.0 % (11) | 16.1 % (9) | 13.7% (7) |  |
| GCS 13–15 | 20.3 % (24) | 22.2 % (22) | 25.5 % (25) | 26.1 % (24) | 16.3 % (14) | 23.1 % (15) | 23.7 % (18) | 30.3 % (20) | 23.9 % (17) | 21.7 % (15) | 29.8 % (25) | 18.8 % (16) | 18.3 % (13) | 14.0% (8) | 15.4 % (12) | 22.4 % (13) | 17.9 % (10) | 17.6% (9) |  |
| Unresponsive pupil/s | 20.3 % (24) | 22.2 % (22) | 16.5 % (16) | 12.2 % (11) | 24.4 % (20) | 27.4 % (17) | 14.7 % (11) | 20.3 % (13) | 26.1 % (18) | 16.2 % (11) | 21.7 % (18) | 21.2 % (17) | 27.1 % (19) | 17.5 % (10) | 20.5 % (16) | 19.3 % (11) | 10.7 % (6) | 32.0% (16) |  |
| Unfavorable Outcome | 43.2% (51) | 44.4% (44) | 52.0% (51) | 40.2% (37) | 55.8% (48) | 46.2% (30) | 47.4% (36) | 36.4% (24) | 50.7% (36) | 46.4% (32) | 52.4% (44) | 35.3% (30) | 47.9% (34) | 47.4% (27) | 52.6% (41) | 41.4% (24) | 51.8% (29) | 64.7% (33) |  |
| 30-Day Mortality | 13.6% (16) | 18.2% (18) | 15.3% (15) | 9.8% (9) | 17.4% (15) | 9.2% (6) | 11.8% (9) | 10.6% (7) | 11.3% (8) | 11.6% (8) | 14.3% (12) | 8.2% (7) | 11.3% (8) | 7.0% (4) | 20.5% (16) | 19.0% (11) | 21.4% (12) | 23.5% (12) |  |
| n total | 118 | 99 | 98 | 92 | 86 | 65 | 76 | 66 | 71 | 69 | 84 | 85 | 71 | 57 | 78 | 58 | 56 | 51 |  |

Table showing Karolinska University Hospital (KUH) patients within different Glasgow Coma Scale (GCS) categories as well as pupil responsiveness, 6-12 months unfavorable outcome and 30-day mortality, stratified by year.

Supplementary Table 3 – Differences per year for patients managed at OUH

| **OUH** | **2015** | **2016** | **2017** | **2018** | **2019** | **2020** | **2021** | **2022** |
| --- | --- | --- | --- | --- | --- | --- | --- | --- |
| Age | 55.5 | 54.7 | 53.9 | 56.4 | 56.8 | 56.3 | 58.6 | 59.3 |
| GCS 3–8 | 46.2 % (97) | 41.3 % (100) | 44 % (88) | 35.5 % (82) | 37.0 % (81) | 38.6 % (93) | 37.5% (94) | 41 % (89) |
| GCS 9–12 | 20.5 % (43) | 16.9 % (41) | 22.5 % (45) | 26.4 % (61) | 21.9 % (48) | 22 % (53) | 19.1 % (48) | 26.7 % (58) |
| GCS 13–15 | 33.3 % (70) | 41.7 % (101) | 33.5 % (67) | 38.1 % (88) | 41.1 % (90) | 39.4 % (95) | 43.4 % (109) | 32.3 % (70) |
| Unresponsive pupil/pupils | 15.2 % (32) | 13.3 % (32) | 12.5 % (25) | 10.4 % (24) | 7.3 % (16) | 12.6 % (30) | 12.8 % (32) | 14.6 % (31) |
| Unfavorable Outcome | 45.7% (96) | 36.8% (89) | 36.5% (73) | 36.8% (85) | 38.4% (84) | 38.2% (92) | 45.8% (115) | 49.3% (107) |
| 30-Day Mortality | 18.6% (39) | 16.9% (41) | 15.0% (30) | 17.3% (40) | 13.7% (30) | 17.8% (43) | 19.9% (50) | 18.0% (39) |
| n total | 210 | 242 | 200 | 231 | 219 | 241 | 251 | 217 |

Table showing Oslo University Hospital (OUH) patients within different Glasgow Coma Scale (GCS) categories as well as pupil responsiveness, 6-12 months unfavorable outcome and 30-day mortality, stratified by year.

*Supplementary Table 4 - Management differences in GCS 3-8 patients*

| **Intervention:** | **All** | **HUS** | **KUH** | **OUH** |
| --- | --- | --- | --- | --- |
| **ICP Monitoring** | 52.5% (n=1378) | 29.3% (n=308) | 74.9% (n=637) | 59.8% (n=433) |
| **Craniotomy** | 49.2% (n=1292) | 50.3% (n=529) | 63.9% (n=543) | 30.4% (n=220) |
| **Craniectomy** | 9.3% (n=244) | 7.8% (n=82) | 12.8% (n=109) | 7.5% (n=54) |
| **Mechanical Ventilation** | 88.2% (n=2314) | 87.9% (n=924) | 99.1% (n=842) | 75.6% (n=548) |
| **Any Neurosurgery** | 70.7% (n=1856) | 65.4% (n=687) | 82.7% (n=703) | 64.2% (n=465) |

Intervention patterns in patients with severe TBI (GCS 3-8) across hospitals. ICP = Intracranial pressure. HUS = Helsinki University Hospital, KUH = Karolinska University Hospital, OUH = Oslo University Hospital.

Supplementary Table 5 - Management differences in Marshall CT 5-6 patients

| **Intervention:** | **Combined** | **HUS** | **KUH** |
| --- | --- | --- | --- |
| **ICP Monitoring** | 35.1% (n=585) | 16.8% (n=185) | 71% (n=399) |
| **Craniotomy** | 77.5% (n=1291) | 76.7% (n=847) | 79.2% (n=445) |
| **Craniectomy** | 7.4% (n=123) | 4.4% (n=49) | 13.2% (n=74) |
| **Mechanical Ventilation** | 90.5% (n=1508) | 90.6% (n=1000) | 90.4% (n=508) |
| **Any Neurosurgery** | 83% (n=1383) | 80.9% (n=893) | 87.2% (n=490) |

Intervention patterns in patients with severe Marshall CT scores (5 - 6) across hospitals. ICP = Intracranial pressure, CT = Computerized Tomography, HUS = Helsinki University Hospital, KUH = Karolinska University Hospital.
